# Notes on Current Neurology

**Published:** 1907-08-31

**Authors:** Purves Stewart


### Notes on Current Neurology.

By PURVES STEWART, M.D., F.R.C.P.

### NEURASTHENIA.

In neurasthenia proper, distinguished from psychasthenia, we have, as Raymond has recently emphasised,* a depression of the whole nervous system, consisting of "irritable feebleness," together with asthenia of all the nervous functions, psychical, sensory, motor, vaso-motor, and trophic. The symptoms of neurasthenia are therefore multiple. They are mainly subjective, though certain objective signs can also be recognised. Charcot long ago pointed out certain neurasthenic "stigmata," some of which are of major importance—*e.g.*, insomnia, headache, neuro-muscular asthenia, spinal pain, gastro-intestinal dyspepsia, mental depression, etc., whilst others are secondary and less constant, such as vertigo, asthenopia, affections of hearing, sensory disturbances, and various circulatory, respiratory, vaso-motor and genital disorders.

The mental state of the neurasthenic patient is one of psychical depression, characterised chiefly by rapid fatigue and feebleness of attention, so that sustained mental effort becomes impossible. Memory, however, is not weakened; but the patient's intellectual activity is diminished, he becomes excessively emotional, loses his normal stoicism and self-control, and develops vague fears and apprehensions. But a point of primary importance, which marks off neurasthenia from psychasthenia and from hysteria, is that there is no perversion of the patient's powers of judgment.

True neurasthenia is not a primary disease. It is the result of something else. The commonest antecedent is over-stress, physical or mental, especially the latter. Accessory causes may also be present—for example, exogenous poisons like morphia, cocaine, tobacco, alcohol, lead; or endogenous toxins such as the sequelæ of infective diseases, especially influenza, malaria, enteric fever, and so on; or poisons actually produced within the body—as by gout and diabetes. Neurasthenia may also be the result of various organic diseases, not only of the nervous system (tabes, disseminated sclerosis, etc.), but also of other systems, as, for example, chronic rheumatism, arterio-sclerosis, the various viscero-ptoses, etc. Lastly, traumatism, as in railway and other accidents, is a well-recognised cause of neurasthenia.

It is possible for neurasthenia to develop in a previously healthy individual as a result of over-strain, mental or physical. But we should note that there is a type of individual who is specially prone to neurasthenia—for example, the "arthritic" type of French writers—whilst on the other hand some individuals, not necessarily of stronger physique, are particularly resistant, and even though subjected to severe strain or to grave organic diseases, such individuals have a coefficient of nervous resistance so high that they do not become neurasthenic.

Clinically, for purposes of treatment, neurasthenic patients can be divided into two classes—namely, those with increased arterial tension and those with vascular hypo-tension. The neurasthenia accompanied by hyper-tension, according to Fleury, is generally the result of some intoxication. In such cases treatment should be directed towards eliminating the toxin by diuretic and laxative drugs, by light diet, encouraging cutaneous elimination by means of baths, exercises, massage, etc. On the other hand, when arterial hypo-tension is present, we must endeavour to augment the nervous energy by specially nourishing diet, by stimulating baths and douches, by massage and electricity. In both classes of cases we must at the same time soothe the patient's nervous irritability and depression by moral treatment—psycho-therapeutics, a factor whose importance cannot be overestimated.

* Bulletin Medical. March 20, 1907.

